# Lernen und Lehren mit digitalen Medien: Eine Standortbestimmung

**DOI:** 10.1007/s11618-021-01047-y

**Published:** 2021-09-29

**Authors:** Katharina Scheiter

**Affiliations:** 1 Arbeitsgruppe Multiple Repräsentationen, Leibniz-Institut für Wissensmedien, Schleichstraße 6, 72076 Tübingen, Deutschland; 2 Universität Tübingen, Tübingen, Deutschland

**Keywords:** Digitale Medien, Unterrichtsqualität, Orchestrierung, Professionelle Kompetenzen von Lehrpersonen, TPACK, Digital media, Orchestration, Professional competences of teachers, Teaching quality, TPACK

## Abstract

Im Beitrag wird der Stand der Forschung zum Einsatz digitaler Medien im Bildungskontext vor dem Hintergrund zweier Forschungsperspektiven beschrieben. Die erste, schon länger vertretene Forschungsperspektive beschäftigt sich mit dem Lernen mit digitalen Medien. Untersucht werden hier vor allem Lernprozesse und -ergebnisse in Abhängigkeit von Medienmerkmalen und individuellen Eigenschaften der Lernenden. Diese Forschungstradition hat zahlreiche Erkenntnisse hervorgebracht. Allerdings wird die Frage nach einer lernwirksamen Integration digitaler Medien in den Unterricht hier nicht berücksichtigt. Hier greift die zweite, jüngere Forschungsperspektive zum Lehren mit digitalen Medien, in welchem das Unterrichten mit digitalen Medien und dafür notwendige professionelle Kompetenzen von Lehrpersonen adressiert werden. Im Beitrag wird abschließend dafür plädiert, beide Perspektiven stärker miteinander zu kombinieren, insbesondere da diese relevante Gemeinsamkeiten in ihren Grundannahmen aufweisen. So werden in beiden Perspektiven digitale Medien weniger anhand von Oberflächenmerkmalen, sondern vielmehr bezüglich ihrer Funktionen für die Förderung von Lern- und Lehrprozessen analysiert.

## Abstract (en)

This article describes the current state of research on the use of digital media in educational contexts against the background of two research perspectives. The first research perspective, which has been established for some time, deals with technology-enhanced learning. It primarily examines learning processes and outcomes as a function of media characteristics and individual learner characteristics. This research tradition has produced numerous findings. However, the question of how to integrate digital media into the classroom in a way that is effective for learning is not considered here. This is where the second, more recent research perspective on technology-enhanced teaching comes in, which addresses teaching with digital media and the professional competencies of teachers required for this. Finally, the paper argues for combining both research perspectives, especially since they have relevant commonalities in their basic assumptions. Thus, in both research perspectives, digital media are analyzed less on the basis of surface features, but rather in terms of their functions for the promotion of learning and teaching processes.

## Einleitung

Die zunehmende Digitalisierung durchdringt alle wesentlichen gesellschaftlichen Bereiche und beeinflusst auch das Bildungswesen in noch nie dagewesener Weise. Zum einen wird Digitalisierung selbst zum Gegenstand von Bildung. Lernende sollen zu einer kompetenten Nutzung digitaler Medien in ihrem Alltag und (späterem) Berufsleben als Voraussetzung für gesellschaftliche Teilhabe befähigt werden. Dies umfasst den Erwerb von Medienkompetenz sowohl für die Nutzung fachunabhängiger digitaler Angebote wie z. B. die kompetente Suche im Internet nach zielrelevanten Informationen als auch für die Anwendung von fachspezifischen digitalen Werkzeugen wie z. B. die Verwendung computerbasierter Tabellenanwendungen und Simulationen für die Lösung mathematischer Fragestellungen. Medienkompetenz beschränkt sich dabei nicht auf technische Bedienfertigkeiten, sondern bezieht sich auch auf eine kritisch-reflektierte Nutzung digitaler Angebote im Hinblick auf damit verbundene Chancen und Risiken für die eigene Person. Für das Bildungspersonal bedeutet diese Erweiterung von Bildungszielen um Medienkompetenz, dass es einerseits selbst über diese Kompetenzen verfügen muss und dass es andererseits Kompetenzen für die Vermittlung von Medienkompetenz aufweisen muss. Hinsichtlich der normativen Frage, ob Medienkompetenz ein relevantes Bildungsziel im 21. Jahrhundert darstellt, herrscht angesichts der Allgegenwärtigkeit des digitalen Wandels Konsens (vgl. Kultusministerkonferenz 2016).

Die zunehmende Digitalisierung verändert Bildung aber auch im Hinblick auf die Gestaltung ihr zugrundeliegender Lehr- und Lernprozesse. Hier steht die Frage im Vordergrund, wie digitale Medien so eingesetzt werden können, dass sie das Erreichen fachlicher und überfachlicher Bildungsziele erleichtern und verbessern. Die Nutzung digitaler Medien dient hier weniger dem Selbstzweck, sondern ist mit der Erwartung eines Mehrwerts für Lehren und Lernen verknüpft. Dieser Mehrwert konnte in diversen Studien belegt werden. Z. B. ergibt eine Zusammenfassung von 25 Meta-Analysen einen kleinen bis mittleren Effekt zugunsten computerbasierter Lernmedien wenn auch bei sehr starken Schwankungen zwischen den einzelnen Metaanalysen (Tamim et al. 2011). Chauhan (2017) findet in einer Meta-Analyse bezogen auf den Elementarbereich basierend auf 122 Studien mit 32.096 Lernenden einen mittleren Effekt zu Gunsten des Lernens mit digitalen Medien (Hedges’ g = 0,55). Fachbezogene Differenzierungen zeigen Schwankungen der Effektgrößen auf, mit großen Effekten für naturwissenschaftliche Inhalte, mittleren Effekten für Sprachen und Mathematik und den kleinsten Effekten für sozialwissenschaftliche Inhalte. Eine aktuelle Meta-Analyse auf der Basis von 92 Studien von Hillmayr und Kollegen (Hillmayr et al. 2020) zeigt, dass der Einsatz digitaler Werkzeuge im mathematischen und naturwissenschaftlichen Unterricht einen positiven mittleren bis großen Effekt auf die Lernleistung hat, wenn man ihn mit dem Unterricht derselben Inhalte ohne Technologie vergleicht (Hedges’ g = 0,65). Zusammenfassend kann die Frage nach dem Mehrwert digitaler Medien auf der Basis dieser Studien also positiv beantwortet werden.

Die Rolle digitaler Medien im Bildungswesen als Lehr-Lernwerkzeug steht im vorliegenden Beitrag im Vordergrund. Ziel ist es, eine Übersicht über die Grundannahmen und -befunde der entsprechenden Forschung zu geben und so eine Verortung von Studienergebnissen in den Gesamtkontext zu erleichtern sowie zukünftige Forschungsbedarfe aufzuzeigen. Der Beitrag konzentriert sich dabei sensu Helmke (2012) auf die Angebotsseite des Unterrichtsgeschehens, d. h. auf das Unterrichtshandeln von Lehrpersonen sowie die eingesetzten Lernmedien.

Im Folgenden werden dabei zwei Forschungstraditionen unterschieden. Bereits in den sechziger Jahren des letzten Jahrhunderts etablierte sich die Forschung zum Lernen mit digitalen Medien (*technology-enhanced learning*, TEL). Hier wird die individuelle Nutzung computerbasierter Lernumgebungen und deren Beeinflussung durch Gestaltungsmerkmale (z. B. Multimedialität, Interaktivität, Vorhandensein von Feedback) und Eigenschaften der Lernenden (z. B. Vorwissen, selbstregulative Fertigkeiten) fokussiert. Der Lernprozess erfolgt in einem in sich abgeschlossenen Szenario, in dem die Interaktion des Lernenden mit dem Medium im Vordergrund steht. Lehrende sind in den entsprechenden Untersuchungszenarien entweder gar nicht anzutreffen oder ihr Handeln wird konstant gehalten und beschränkt sich auf die Administration der computerbasierten Instruktion. Das Medium bestimmt hier die Gesamtheit des instruktionalen Geschehens, dessen Lernwirksamkeit entsprechend auf einzelne Medien und deren spezifische Gestaltung zurückgeführt wird. Diese Forschungsperspektive ist im deutschsprachigen Raum traditionell eher der experimentell arbeitenden Lehr-Lernforschung zuzuordnen.

Diese Tradition wird spätestens seit der Jahrtausendwende um eine Forschungsperspektive ergänzt, die sich mit der Frage des Lehrens mit digitalen Medien (*technology-enhanced teaching*, TET) auseinandersetzt. Hier werden digitale Medien als Bestandteil eines komplexen Lehr-Lernarrangements verstanden, welches durch die Gesamtheit unterschiedlicher (digitaler und analoger) Angebote im Unterricht gekennzeichnet ist. Der Fokus rückt auf den Unterrichtskontext, in den digitale Medien eingebettet und mit anderen Angeboten verknüpft werden (als Orchestrierung bezeichnet), sowie auf die Rolle der Lehrenden, die diesen Kontext planen und umsetzen. Entsprechend werden Lernergebnisse von Schülerinnen und Schülern auf den Unterricht mit digitalen Medien zurückgeführt und durch die dort stattfindenden Lehr- und Lernprozesse erklärt, während bei TEL lediglich Lernprozesse als erklärende Variablen Berücksichtigung finden. TET ist insofern als die jüngere der beiden Perspektiven zu betrachten als dass die entsprechende Forschung erst mit einer nennenswerten Verfügbarkeit digitaler Medien in Schulen möglich wurde, während die TEL-Forschung in laborexperimentellen Settings ihren Ursprung hat.

Sowohl die TEL- als auch die TET-Perspektive werden im Folgenden beschrieben, bevor Implikationen für zukünftige Forschung gezogen werden. Diese greifen ein Plädoyer eines der Pioniere der Medienforschung, Gavriel Salomon, auf, der bereits 1990 forderte, dass medienbezogene Forschung sich sowohl mit den Medien als auch mit deren Einbettung in den Unterricht auseinandersetzen müsse – „studying the flute and the orchestra“ (Salomon 1990, S. 521).

## Lernen mit digitalen Medien (TEL) – Wirkungen und Affordanzen

Digitale Medien werden seit Beginn der sechziger Jahre des letzten Jahrhunderts für die Wissensvermittlung genutzt. Anfangs standen viele digitale Anwendungen noch in der Tradition programmierten Unterrichts (vgl. Skinner 1986). Das heißt, digitale Medien ermöglichen es Lernenden, eng umschreibbare Fertigkeiten wie die Ausführung der Grundrechenarten einzuüben und durch Vergabe computerbasierten Feedbacks kontinuierlich zu verbessern und deren Ausführung zu automatisieren. Technische Innovationen haben dazu geführt, dass die Möglichkeiten der Gestaltung und Nutzung digitaler Lernumgebungen sich seitdem ständig erweitert haben – von rein symbolisch-abstrakten Darbietungsformen hin zu multimedialen, immersiven Angeboten (z. B. virtuelle Realitäten), von statischen und linearen Strukturen hin zu durch Lernende manipulierbare und vernetzte Informationsangebote (z. B. Simulationen, Hypermedia), von der medial vermittelten Präsentation vorgegebener Informationen hin zu konstruktiv einsetzbaren Werkzeugen, mit deren Hilfe Lernende ihre Lernumgebung selbst gestalten oder auch von ausschließlich individuell nutzbaren hin zu kollaborativen Lernumgebungen, in denen Lernende sich austauschen und gemeinsam an Artefakten arbeiten können. Die Forschung zu diesen digitalen Lernumgebungen wird in der angloamerikanischen Literatur unter dem Begriff „technology-enhanced learning“ zusammengefasst, um der Annahme Rechnung zu tragen, dass damit eine Erweiterung bisheriger *Lern*möglichkeiten verbunden ist.

Allerdings ist die mit TEL verbundene Medienwirkungsforschung bereits seit ihren Anfängen massiv umstritten, da sie zu stark auf die Technologie und zu wenig auf den Lernprozess bezogen ist (Zinger et al. 2017). Der prominenteste Kritiker Richard Clark (1983) geht dabei davon aus, dass es sich bei den oben beschriebenen Effekten um das Ergebnis einer Konfundierung von Medium und Instruktionsmethode handelt. Nach Clark sind Medien lediglich Informationsträger, die als solche keinen Effekt auf Lernen haben: „media are mere vehicles that deliver instruction but do not influence student achievement any more than the truck that delivers our groceries causes changes in our nutrition“ (Clark 1983, S. 445). Dem entgegnet Robert Kozma (1991, 1994), dass es sich dabei nicht um eine Konfundierung handelt, die bei sorgfältiger Studienplanung aufgelöst werden könnte. Vielmehr weisen digitale Medien inhärente Eigenschaften bzw. Funktionalitäten für das Lernen auf, die untrennbar mit ihnen verbunden sind. Positive Lerneffekte ergeben sich danach dann, wenn eine Instruktionsmethode sich diese Funktionalitäten so zu Nutzen macht, dass damit aufgabenrelevante kognitive Verarbeitungsprozesse unterstützt werden, die ohne das Medium in dieser Weise nicht möglich wären. Die Clark-Kozma Debatte hat nichts an Aktualität verloren: Die in der öffentlichen Debatte oftmals diskutierte Frage nach der Wirksamkeit digitaler Medien („Helfen Computer beim Lernen?“) ist in Einklang mit Clark (1983) ebenso wenig zielführend wie die Durchführung von globalen Medienvergleichsstudien (Lernen mit vs. ohne Computer) zu ihrer Beantwortung, da sie eine fehlerhafte Attribution möglicher Effekte nahelegen. Dass entsprechende Studien aber trotzdem Effekte zeigen, liegt in Einklang mit Kozma (1991, 1994) daran, dass hier bereits Medien so genutzt werden, dass ihre Funktionalitäten lernwirksam werden können.

Mit ausgelöst durch die Clark-Kozma Debatte ist die TEL-Forschung daher zunehmend durch die Frage geprägt, welche Medieneigenschaften geeignet sind, um lernbezogene kognitive Prozesse anzuregen, deren Ausführung zu unterstützen und zu erleichtern (Gerjets und Hesse 2004). Um lernwirksame digitale Lernmedien zu entwickeln, muss also zunächst identifiziert werden, welche Medieneigenschaften eine Passung zu für ein bestimmtes Lernziel als relevant erachteten Lernprozessen aufweisen. Diese können als Affordanzen beschrieben werden. Hierbei handelt es sich um ein Konzept aus der ökologischen Psychologie (Gibson 1977), welches von Don Norman (1988) auf die Gestaltung digitaler Schnittstellen übertragen wurde. Danach haben Objekte bzw. gute Designs einen ihnen inhärenten Aufforderungscharakter (Affordanz), der dazu führt, dass mit diesem Objekt automatisch entsprechend interagiert oder gehandelt wird. Übertragen auf das Lernen mit digitalen Medien gibt es Medieneigenschaften wie z. B. die Möglichkeit zur Darstellung dynamischer Abläufe in einer Animation, die einen lernrelevanten kognitiven Prozess z. B. das Nachvollziehen der Veränderung des Darstellten (z. B. die Veränderung des Zellkerns während der Zellteilung) auslösen können. Entsprechend zeigen Studien, dass beim Einsatz digitaler Medien das Passungsverhältnis zwischen Affordanz (Medieneigenschaft) und Lernprozess bzw. Lernziel entscheidend ist. So zeigt eine Meta-Analyse basierend auf 194 Studien zum Lernen mit dynamischen Visualisierungen (Ploetzner et al. 2020), dass diese im Vergleich zu statischen Visualisierungen nur dann lernförderlich sind, wenn das Nachvollziehen eines prozesshaften Geschehens (z. B. das Verstehen der Kontinuität der Veränderung) als Lernziel im Vordergrund steht. Ebenso zeigen sich besonders deutliche Effekte digitaler Medien, wenn diese individuell an die Lernprozesse von Schülerinnen und Schülern angepasst sind so wie dies der Fall für adaptive Lernsysteme wie z. B. intelligente tutorielle Systeme der Fall ist (Aleven et al. 2017). Intelligente tutorielle Systeme basieren auf einer vollständigen Modellierung des Gegenstandsbereichs, indem zunächst für die Aufgabenbearbeitung notwendige deklarative und prozedurale Wissensbestände spezifiziert werden (Aufgabenmodell). Für jeden Lernenden wird dann ein Lernermodell aus Aufgabenlösungen und Fehleranalysen generiert. Dieses Lernermodell wird mit dem Aufgabenmodell kontinuierlich abgeglichen, um Defizite im Wissensbestand zu identifizieren, die dann durch adaptives Feedback und Erklärungen adressiert werden. Eine Meta-Analyse basierend auf 107 Effektgrößen und 14.321 Lernenden von Ma et al. (2014) bestätigt die Lernwirksamkeit intelligenter tutorieller Systeme gegenüber regulärem Unterricht durch eine Lehrperson (Hedges’ g = 0,42), anderen computerbasierten Lernanwendungen (Hedges’ g = 0,57) oder dem Lernen mit Büchern und Arbeitsheften (Hedges’ g = 0,35). Diese Effekte verschwinden gegenüber Lernszenarien, in denen eine Lehrperson für einen Lernenden bzw. für eine kleine Gruppe von Lernenden verantwortlich ist. Hier kann (technologiefreier) Unterricht in ähnlichem Ausmaß adaptiv gestaltet werden wie durch ein intelligentes tutorielles System, so dass beide Ansätze funktional äquivalent sind (Corno 2008; Dumont 2019). Zusammenfassend muss eine globale Medienwirkungsforschung also durch eine Forschung zu Affordanzen digitaler Medien und deren spezifischen lernrelevanten Funktionen abgelöst werden.

Eine weitere Kritik an der Medienwirkungsforschung bezieht sich auf die unzureichende Spezifikation der Effekte. Nach Salomon und Perkins (2005) müssen Effekte mit Medien (*effects with media*) von Effekten von Medien (*effects of media*) unterschieden werden. Effekte mit Medien sind während des Medieneinsatzes zu beobachten und auf diesen Zeitraum beschränkt. Mit einem Taschenrechner kann eine Person mathematische Aufgaben besser lösen, aber nur so lange sie den Rechner nutzt. Effekte von Medien sind dagegen dadurch gekennzeichnet, dass sie auch dann noch zu beobachten sind, wenn das Medium nicht mehr verfügbar ist. Auswirkungen des Lernens mit digitalen Medien auf den Wissenserwerb sollten auch dann noch nachweisbar ist, wenn das Medium keine weitere Unterstützung mehr bietet. Die von Salomon und Perkins eingeführte Unterscheidung legt darüber hinaus nahe, dass Wirkungen des Lernens mit digitalen Medien nicht nur kurzfristig und auch im Hinblick auf andere Kriterien untersucht werden sollten. Adaptive Lerntechnologien, die den Lernstand des Lernenden automatisch diagnostizieren und darauf angepasst Feedback geben (Aleven et al. 2017), können nicht nur den Wissenserwerb beeinflussen, sondern möglicherweise auch die Selbstregulationsfähigkeiten des Lernenden. Lernende könnten zum einen durch das unmittelbare Feedback in die Lage versetzt werden, ihren Lernprozess akkurater einzuschätzen und zu regulieren und damit bessere Selbstregulationsfähigkeiten erwerben, die sich auch in Lernsituationen ohne adaptives Lernsystem nachhaltig positiv auswirken. Adaptive Lerntechnologien könnten aber auch dazu führen, dass Lernende Selbstregulationsfähigkeiten „verlernen“, indem sie abhängig von der Rückmeldung des Systems werden. Solche Wirkungen von Medien lassen sich nur in Studien untersuchen, in denen Lernen mit digitalen Medien über einen längeren Zeitraum und auch bezogen auf mögliche (positive und negative) Nebenwirkungen hin untersucht wird. Klassische Medienwirkungsstudien sind aber oftmals auf kurzfristige Effekte auf den Wissenserwerb beschränkt.

Schließlich bezieht sich der Großteil der TEL-Forschung auf Lernszenarien, in denen digitale Werkzeuge bestenfalls ergänzend zu, aber häufiger vollständig unabhängig von dem sonstigen Lehr-Lerngeschehen eingesetzt werden (Tamim et al. 2011). Das heißt, die betrachtete Lernsituation beschränkt sich auf die Interaktion des Lernenden mit dem digitalen Medium, ohne dass die Mediennutzung in den Unterrichtskontext eingebunden ist. Das bedeutet auch, dass Lehrpersonen in diesen Studien entweder gar nicht anwesend sind oder nur eine sehr eingeschränkte Rolle einnehmen, indem sie beispielsweise die Studienabläufe mit koordinieren. Salomon fordert bereits 1990 eine Änderung dieses analytischen Forschungsansatzes zu Gunsten einer systemischen Betrachtung der Wirkungen digitaler Medien unter Berücksichtigung individueller, sozialer und kultureller Randbedingungen: „the age of computers-*in*-education (to be distinguished from computer *and* education) challenges us with a need to consider relatively new research paradigms to complement the ones that we are familiar with“ (Salomon 1990, S. 522). Entsprechend diskutieren Anderson, Corbett, Koedinger und Pelletier (1995) die Abstimmung auf den Unterricht als wesentlich für den Erfolg der von ihnen entwickelten kognitiven Tutoren. Insbesondere ist es entscheidend, Lehrpersonen auf das Unterrichten mit den kognitiven Tutoren durch intensive Fortbildungen vorzubereiten sowie eine Passung der Tutoren mit dem eigentlichen Curriculum herzustellen. Ebenso zeigt auch eine Metaanalyse von Hillmayr et al. (2020) dass der Einsatz digitaler Medien im naturwissenschaftlichen Domänen einen stärkeren Effekt auf Schülerleistungen hat, wenn die Lehrpersonen im Umgang mit der Technologie geschult worden waren (Hedges’ g = 0,84) als wenn dies nicht der Fall war (Hedges’ g = 0,56).

Vor diesem Hintergrund rückt die Frage in den Vordergrund, wie guter Unterricht mit digitalen Medien (*technology-enhanced teaching*) gestaltet sein muss und welche Kompetenzen Lehrpersonen dafür aufweisen müssen (vgl. Zinger et al. 2017).

## Lehren mit digitalen Medien (TET) – Wirkungen und Orchestrierung

Auf konzeptueller Ebene ist die TET-Perspektive durch eine Reihe von Modellen geprägt, die auch als Medienintegrationsmodelle bezeichnet werden (z. B. Niederhauser und Lindstrom 2018). Allerdings konzentrieren sich die so zusammengefassten Modelle auf sehr unterschiedliche Aspekte von TET. Zum einen gibt es Modelle, die sich mit der Frage beschäftigen, welche Formen der Mediennutzung im Unterricht mit einem Mehrwert gegenüber herkömmlichem Unterricht verbunden sind. Die entsprechenden Modelle zur Funktion von Medien im Unterricht werden im Abschn. 3.1 beschrieben. Zum anderen fallen unter den Begriff der Medienintegration Modelle, die diejenigen Merkmale von Lehrpersonen beschreiben, welche mit einer häufigeren oder lernwirksameren Mediennutzung im Unterricht einhergehen. Diese werden in Abschn. 3.3 beschrieben. Das Konzept der Orchestrierung (3.2) ermöglicht eine Verknüpfung der beiden Schwerpunkte, indem hervorgehoben wird, dass potenziell lernwirksame Funktionen von Medien vor allem dann zur Geltung kommen, wenn sie sinnvoll mit anderen Funktionen kombiniert werden. Dabei nehmen Lehrpersonen und ihre Kompetenz für das Unterrichten mit digitalen Medien eine zentrale Rolle ein.

### Mehrwert digitaler Medien aus der Unterrichtsperspektive

In gleicher Weise, wie in der Forschung zum Lernen mit digitalen Medien betont wird, dass der Mehrwert digitaler Werkzeuge aus bestimmten funktionalen Eigenschaften (Affordanzen) für Lernprozesse resultiert, wird auch in Modellen zum Lehren mit digitalen Medien betont, dass diese eine funktionale Erweiterung des sonstigen Unterrichtshandelns erlauben müssen. Im RAT-Modell (Hughes et al. 2006) können digitale Medien drei verschiedene Funktionen im Unterricht einnehmen: Sie können bisherige Unterrichtspraktiken, Lernprozesse und Lernziele ersetzen, ohne diese zu verändern (*Replacement*), sie können deren Wirkung verstärken (*Amplification*) oder grundsätzlich verändern (*Transformation*). In ähnlicher Weise argumentiert Puentedura (2006) in seinem SAMR-Stufenmodell, in dem er von vier Modi der Mediennutzung ausgeht: *Substitution, Augmentation, Modification* und *Redefinition*. Die Modifikation und Neugestaltung von Lehr-Lernprozessen und Unterrichtszielen entspricht dabei einer transformativen Nutzung im RAT-Modell von Hughes et al. (2006). Beide Modelle gehen davon aus, dass eine transformative Nutzung digitaler Medien im Unterricht bessere Leistungen von Schülerinnen und Schülern nach sich ziehen sollte als z. B. die Ersetzung bestehender Unterrichtspraktiken. Insbesondere auf das SAMR-Modell wird auch in bildungspolitischen Diskursen sehr häufig Bezug genommen, obwohl es sich eher um eine pragmatische Taxonomie aber keinesfalls um eine empirisch abgesicherte Vorstellung handelt. Puentedura (2014) zieht als Evidenz vier Studien zum Unterrichten mit digitalen Medien heran, die er den vier Stufen des Modells zuordnet und deren Effektgrößen von der untersten Stufe (*Substitution*) zur obersten Stufe (*Redefinition*) zunehmen. Hamilton, Rosenberg und Akcaoglu (2016) kritisieren, dass diese Studien willkürlich ausgewählt wurden. Außerdem fehlen valide Operationalisierungen der Stufen für beide Modelle, die es erlauben würden, diesen unterschiedliche medienbasierte Unterrichtsformen zuverlässig zuzuordnen.

Eine von Lachner et al. (2020; siehe auch Klieme 2020 und Voss 2020, für das Lernen und Lehren auf Distanz) vorgeschlagene Alternative besteht darin, Forschung zum Unterrichten mit digitalen Medien stärker an der klassischen Unterrichtsforschung auszurichten. Danach lässt sich eine hohe Unterrichtsqualität (z. B. Kunter et al. 2013; Lipowsky et al. 2009) an drei Prozessmerkmalen festmachen: (a) effiziente Klassenführung, bei der nicht-aufgabenbezogenes Verhalten zugunsten einer Maximierung der Lernzeit reduziert wird, (b) kognitive Aktivierung durch das Präsentieren von herausfordernden Aufgaben, die für Lernende noch bewältigbar sind, wenn sie richtig gestellt und unterstützt werden (d. h. innerhalb der Zone der proximalen Entwicklung, Vygotski 1987), und (c) ein unterstützendes Klima, in dem sich die Schülerinnen und Schüler wertgeschätzt fühlen und die notwendige Anleitung erhalten, die ihnen beim Lernen hilft. Lernwirksamer Unterricht mit digitalen Medien sollte also durch eine hohe Prozessqualität entlang dieser drei Dimensionen gekennzeichnet sein. Eine empirische Überprüfung dieser Annahme steht jedoch noch aus. Allerdings konnte bereits gezeigt werden, dass Schülerinnen und Schüler, die über ein Schulhalbjahr mit Tabletcomputern unterrichtet wurden, die konstruktive Unterstützung dieses Unterrichts positiver bewerteten als Schülerinnen und Schüler aus Kontrollklassen ohne Tabletcomputer (Hammer et al. 2021). Dies galt insbesondere für diejenigen Schülerinnen und Schüler, die über ungünstigere Eingangsvoraussetzungen verfügten (z. B. geringere Motivation, schwächere kognitive Leistungsfähigkeit). Diese Befunde können als erste Evidenz für einen positiven Zusammenhang zwischen medienbasiertem Unterricht und dessen Prozessqualität interpretiert werden. Offen ist bislang die Frage, was genau diese höhere Prozessqualität bedingt und ob es einen Zusammenhang mit den Lernergebnissen der Schülerinnen und Schüler gibt.

Eine Anbindung der TET-Forschung an die Unterrichtsforschung hat mindestens zwei Vorteile: Erstens existieren in der Unterrichtsforschung diverse Befunde und Methoden zur Erfassung der Prozessqualität von Unterricht, die als Ausgangspunkt für die TET-Forschung genutzt werden können. Zweitens rückt damit der Fokus auf das gesamte (mediengestützte und analog umgesetzte) Unterrichtsgeschehen, während im RAT- und SAMR-Modell immer impliziert wird, dass zunächst ein analoges Vorgehen existiert, welches dann durch den Medieneinsatz ersetzt, erweitert oder transformiert wird. Diese Sichtweise, die sich auch in dem Begriff der Digitalisierung widerspiegelt (Verwandlung von etwas nicht-Digitalem in etwas Digitales), wird zunehmend kritisch gesehen, da es sich bei der Gestaltung medienbasierten Unterrichts eben nicht lediglich um die Übersetzung analogen Unterrichts handelt bzw. handeln sollte. Auch wegen des zunehmenden Verschmelzens der Grenzen zwischen digital und analog setzt sich aus diesem Grund im deutschsprachigen Raum immer mehr der Begriff der Digitalität durch, der keinen „analogen Vorgänger“ impliziert.

### Orchestrierung des Unterrichts mit digitalen Medien

Während sich das oben beschriebene RAT-Modell und das SAMR-Modell auf die Frage konzentrieren, wann Medien einen funktionalen Mehrwert für den Unterricht mit sich bringen, bezieht sich der Begriff der Orchestrierung auf den Prozess der Einbindung digitaler Medien in das komplexe Unterrichtsgeschehen (Dillenbourg 2013; Sharples 2013). Mit der Forderung nach einer didaktisch wirksamen Orchestrierung ist die Auffassung verbunden, dass der Erfolg mediengestützten Unterrichts nicht nur durch die Qualität der Lernmedien bedingt wird. Diese müssen vielmehr in Abhängigkeit von Lernziel, kontextuellen Gegebenheiten, angestrebter Sozialform des Unterrichts und den jeweiligen Funktionen des Lehr-Lernangebots zu einer Choreographie des Unterrichts beitragen, wobei unterschiedliche Choreographien für das gleiche Medium denkbar sind (Oser und Baeriswyl 2001). Beispielsweise kann das Anfertigen einer digitalen Mindmap in Kleingruppen zu Beginn einer Unterrichtseinheit der Vorwissensaktivierung dienen, während am Ende einer Unterrichtseinheit damit auf eine Konsolidierung des Wissens abgezielt wird. Wesentlich für die Idee der Orchestrierung digitaler Medien ist die Annahme, dass digitale Medien pädagogisch sinnvoll mit anderen (analogen) Unterrichtsangeboten verknüpft werden. Zum Beispiel kann im naturwissenschaftlichen Unterricht forschendes Lernen sowohl mit Realexperimenten als auch mit virtuellen, am Computer simulierten Experimenten umgesetzt werden. Hier zeigt die Forschung, dass beide Formen des Experimentierens aus unterschiedlichen Gründen lernwirksam sind (de Jong et al. 2013). Insbesondere erfüllen Realexperimente und virtuelle Experimente unterschiedliche didaktische Funktionen (z. B. Fokussierung auf die Beobachtung des sichtbaren Phänomens im Realexperiment vs. Möglichkeit der Modellierung des Phänomens auf einem höheren Abstraktionsgrad im virtuellen Experiment). Entsprechend ergibt sich die höchste Lernwirksamkeit durch die Kombination beider Experimentalformen, wenn die Funktionen beider Angebote zusammenwirken (Wörner et al. 2021). So kann die Durchführung des Realexperiments am Anfang mit dem zu untersuchenden Phänomen vertraut machen, welches dann im virtuellen Experiment im Detail untersucht werden kann. Umgekehrt kann die Nutzung eines virtuellen Experiments genutzt werden, um durch eine schrittweise, abstrahierte Einführung des komplexen Zusammenhangs auf die Beobachtung des Phänomens im Realexperiments vorzubereiten.

Mit dem Konzept der Orchestrierung ist die Auffassung verbunden, dass dem Dirigenten/der Dirigentin eine entscheidende Funktion zukommt. Die Lehrperson ist dafür verantwortlich, dass eine didaktisch sinnvolle Choreographie resultiert, in der digitale und analoge Unterrichtsangebote in Harmonie zusammenwirken, und muss dafür eine entsprechende Kompetenz aufweisen.

### Digitalisierungsbezogene professionelle Kompetenz von Lehrpersonen

In Einklang mit Baumert und Kunter (2006) wird die professionelle Kompetenz von Lehrpersonen auch im Kontext medienbasierten Lehrens als Voraussetzung für die Bewältigung von berufsbezogenen Anforderungen verstanden, die sowohl kognitive (Wissen und Fertigkeiten) als auch evaluativ-affektive Aspekte (z. B. motivationale Orientierungen, Einstellungen und Überzeugungen) umfasst (siehe auch Weinert 2001). Die digitalisierungsbezogene Kompetenz von Lehrkräften wird vor allem unter zwei Gesichtspunkten berücksichtigt: zum einen als Prädiktor für die Bereitschaft zur Mediennutzung und zum anderen als Voraussetzung für didaktisch sinnvollen, lernwirksamen Unterricht mit digitalen Medien.

#### Kompetenz als Voraussetzung für Mediennutzung

Das bekannteste Modell, das sich mit der Bereitschaft zur Integration digitaler Medien in den Unterricht bzw. zu deren Nutzung auseinandersetzt, ist das so genannte Will-Skill-Tool Modell von Knezek und Christensen (2016). Danach ist es wahrscheinlicher, dass Lehrpersonen Technologien im Unterricht einsetzen, wenn sie eine positive Einstellung zum Einsatz von Technologie im Unterricht haben (Will), die notwendigen Fertigkeiten besitzen (Skill) und Zugang zu den Technologien haben (Tool). Petko (2012) fand heraus, dass 60 % der Varianz in der Intensität des Einsatzes digitaler Medien für den Unterricht durch die drei Faktoren erklärt wird. Laut Knezek und Christensen (2016) hängt der Vorhersagewert der einzelnen Faktoren allerdings vom tatsächlichen Grad der Technologienutzung in einem Kontext (z. B. Bildungssystem) ab. Eine geringe Nutzung hängt meist mit einem mangelnden Zugang zu digitalen Werkzeugen zusammen, während bei insgesamt häufigerer Mediennutzung motivationale Faktoren am wichtigsten sind. Ein wesentliches methodisches Problem der Forschung im Kontext des Will-Skill-Tool-Modells besteht in der Erfassung von Fertigkeiten über Selbstberichte. Darüber hinaus wurde das ursprünglich für den industriellen Kontext entwickelte Technologie-Akzeptanz-Modell (Technology Acceptance Model [TAM]; Davis 1989), das sich auf Einstellungen gegenüber Technologie konzentriert, auch auf Lehrpersonen angewendet. Nach dem TAM hängt die Verhaltensabsicht, Medien (für den Unterricht) zu nutzen, von den Einstellungen (*attitudes*) gegenüber der Technologie ab, die wiederum von der wahrgenommenen Benutzerfreundlichkeit (*ease of use*) und der wahrgenommenen Nützlichkeit der Technologie (*perceived usefulness*) beeinflusst werden. Scherer und Teo (2019) zeigten in einer Meta-Analyse von 45 Studien, dass 39,2 % der Varianz in den Nutzungsabsichten von Lehrpersonen durch TAM-Variablen erklärt werden können. Insbesondere die wahrgenommene Nützlichkeit der Technologie steht in einem konsistenten Zusammenhang mit den Nutzungsintentionen und zeigt – im Gegensatz zum ursprünglichen TAM-Modell – nicht nur indirekte Effekte (vermittelt über die Einstellungen der Lehrkräfte), sondern auch direkte Effekte auf die Nutzungsintentionen. Eine wesentliche Erkenntnis aus der Forschung zur Nutzung digitaler Medien ist, dass die diesbezügliche Motivation die wesentliche Barriere für die Realisierung medienbasierten Unterrichts darstellt (so genannte *second-order barrier *nach Ertmer et al. 2012), die selbst bei umfassender Verfügbarkeit digitaler Medien (*first-order barrier*) die Integration im Unterricht verhindern kann.

#### Kompetenz als Voraussetzung für lernwirksamen mediengestützten Unterricht

Ein Großteil der Forschung zu digitalisierungsbezogener professioneller Kompetenz fokussiert auf eine Beschreibung und Ausdifferenzierung kognitiver Dimensionen (Wissen) mit dem Ziel der Vorhersage der Unterrichtsqualität. Das TPACK-Modell von Mishra und Koehler (2006) stellt das in der Forschung am häufigsten verwendete Rahmenmodell zur Beschreibung des professionellen Wissens von Lehrerpersonen im Zusammenhang mit mediengestütztem Unterricht dar (Harris et al. 2017; Petko 2020). Petko (2020) stellt fest, dass bis 2019 1008 Publikationen zum TPACK-Modell erschienen sind. Das TPACK-Modell basiert auf der klassischen Konzeption des Wissens von Lehrpersonen von Shulman (1987), welche pädagogisches Wissen (*pedagogical knowledge*, PK), domänenspezifisches Fachwissen (*content knowledge*, CK) und pädagogisches Fachwissen bzw. fachdidaktisches Wissen (*pedagocical content knowledge,* PCK) umfasst. Um den zusätzlichen Anforderungen, die sich aus dem Einsatz digitaler Medien im Unterricht ergeben, Rechnung zu tragen, fügten Mishra und Koehler (2006) dieser Klassifikation technologisches Wissen (TK) als Dimension hinzu. Aus unterschiedlichen Kombinationen von PK, CK und TK werden drei weitere intermediäre Wissensformen postuliert (PCK, TCK, TPK). TPACK ist im Schnittpunkt dieser drei Kombinationen angesiedelt. Nach Mishra und Koehler (2006) bildet TPACK die Grundlage für guten mediengestützten Unterricht und erfordert ein Verständnis dafür, wie Konzepte mit Hilfe digitaler Medien unter Berücksichtigung typischer alternativer Vorstellungen von Schülerinnen und Schüler dargestellt und vermittelt werden können.

Trotz der großen Beliebtheit des TPACK-Modells weist sowohl die Konzeption als auch die darauf bezogene empirische Forschung diverse Mängel auf (zur Kritik siehe z. B. Angeli und Valanides 2009; Graham 2011; Petko 2020; Voogt et al. 2013). Erstens handelt es sich bei TK und den darauf aufbauenden Wissensformen um ein schlecht definiertes Konstrukt mit unscharfen Grenzen zwischen den postulierten Wissenstypen. Mishra und Koehler (2006) definierten TK zunächst als Wissen über Betriebssysteme und Computer-Hardware sowie die Fertigkeit, Software-Tools zu verwenden. Koehler und Mishra (2009) betonen, dass die Definition von TK notorisch schwierig ist, weil digitale Technologien im Gegensatz zu physischen Geräten wie einem Stift auf unterschiedliche Weise verwendet werden können, sich im Laufe der Zeit verändern und undurchsichtig sind, da ihre Funktionsweise für den Benutzer verborgen bleibt. Vor diesem Hintergrund präsentieren sie eine Definition von TK, die eng an den Begriff der Fluency of Information Technology (FITness, vgl. National Research Council 1999) anknüpft. FITness erfordert, dass Personen Informationstechnologie hinreichend gut verstehen, um sie bei der Arbeit und im Alltag produktiv einzusetzen, um zu erkennen, wann Informationstechnologie das Erreichen eines Ziels unterstützen oder behindern kann, und um sich kontinuierlich an Veränderungen in der Informationstechnologie anzupassen. Da diese Definition das Wissen über die Affordanzen einer Technologie für Lehren und Lernen hervorhebt, macht sie TK allerdings untrennbar von technologisch-pädagogischem Wissen (TPK) (Graham 2011).

Darüber hinaus gibt es in der Literatur unterschiedliche Ansichten über die Entstehung von TPACK. Eine integrative Sichtweise, bei der jede einzelne Basiskomponente separat und direkt zum übergeordneten Konstrukt von TPACK beiträgt, wird dabei von einer transformativen Sichtweise unterschieden, bei der dieser Beitrag durch TPK, TCK und PCK vermittelt wird (vgl. Angeli und Valanides 2009; Schmid et al. 2020). Nach der transformativen Sichtweise ist TPACK ein einzigartiges Konstrukt, das sich unabhängig von den grundlegenderen Wissenstypen entwickelt. Wie TPACK entsteht, ist nicht nur aus theoretischer Sicht interessant, sondern hat auch wichtige Implikationen für die Bildungspraxis. Zum Beispiel wäre es nach der integrativen Sichtweise in der Lehrerausbildung ausreichend, jede Basiskomponente zu lehren (d. h. TK zu bestehenden Curricula hinzuzufügen), während nach der transformativen Sichtweise TPACK explizit vermittelt werden muss (Petko 2020).

Schließlich ergeben sich nach dem TPACK-Modell an jedem möglichen Schnittpunkt unterschiedliche Wissenskomponenten, von denen angenommen wird, dass sie bedeutungsvoll sind, gemessen werden können und einen eigenen Beitrag zur Unterrichtsqualität leisten. Empirisch konnte die vorgeschlagene Struktur mit sieben Wissenskomponenten nur in wenigen Studien bestätigt werden (z. B. Lin et al. 2013), während die Mehrzahl der Studien weniger Dimensionen identifiziert hat. Scherer et al. (2017) fanden eine verschachtelte Struktur, die den Zusammenhang zwischen den verschiedenen technologiebezogenen Teilkomponenten am besten beschreibt. Danach gibt es einen allgemeinen Faktor sowie eine TK-Komponente. Jedoch findet sich keine klare Unterscheidung zwischen den übrigen vier TK-bezogenen Komponenten.

Neben diesen konzeptuellen Problemen betrifft die zweite Einschränkung die Messung von TPACK (vgl. Angeli und Valanides 2009; Petko 2020; Seufert et al. 2021). Fast alle Studien verwenden Selbstberichte, um TPACK und seine verschiedenen Teilkomponenten zu beurteilen (vgl. Schmidt et al. 2009). Die Verwendung von Selbstberichten ist problematisch, da viele Forschungsergebnisse zeigen, dass die Urteile von Menschen über ihr Wissen und ihre Leistung oft ungenau sind und mit Verzerrungen behaftet sind (Bjork et al. 2013). Selbstberichte über Wissen und Leistung erfassen daher eher die Selbstwirksamkeitsüberzeugung einer Person, also das Vertrauen in die eigenen Fähigkeiten, eine Aufgabe erfolgreich zu bewältigen (Bandura 1977). Eine Sekundäranalyse von Daten unter anderem aus PIAAC (*Programme for the International Assessment of Adult Competencies*; Teilstichprobe von im Bildungswesen tätigen Personen, 93 % Lehrpersonen, *n* = 2590) zeigt, dass objektive Messungen digitaler Kompetenz (bezogen auf die Nutzung von Technologie zur Bewältigung alltäglicher Aufgaben) nur schwach mit entsprechenden Selbstberichten assoziiert sind (Hämäläinen et al. 2021).

Derzeit gibt es nur wenige Instrumente, die versuchen, tatsächliches digitalisierungsbezogenes Wissen von Lehrpersonen zu messen. Diese erfassen fachunabhängig technologisch-pädagogisches Wissen (TPK). Lachner et al. (2019) haben einen Test zur Messung von TPK entwickelt, der Multiple-Choice-Fragen zur Erhebung des zugrundeliegenden konzeptionellen Wissens und kurze textbasierte Vignetten zur Messung von situativem TPK verwendet. Eine erste Studie zeigte, dass das Instrument geeignet ist, zwischen verschiedenen Erfahrungsniveaus von Lehrenden zu unterscheiden. In einer zweiten Studie ergaben sich Zusammenhänge zwischen TPK mit dem pädagogischen Wissen der Teilnehmenden, aber nicht mit ihrem technologischen Wissen. In einer Studie im Kontext des Home Schoolings während der COVID-19-Pandemie von König et al. (2020) sagte die Subdimension konzeptuelles Wissen erfolgreich vorher, ob sich die Lehrpersonen in der Lage fühlten, den Kontakt zu ihren Schülerinnen und Schülern aufrechtzuerhalten und eine Binnendifferenzierung vorzunehmen, was die Validität des Tests weiter bestätigt. Baier und Kunter (2020) schlugen ein TPK-Instrument mit offenen Items vor. Hier werden Lehrpersonen aufgefordert, technologische Affordanzen auf pädagogische Funktionen abzubilden (z. B. „Sie als Lehrer möchten Ihren Unterricht an die individuellen Lernbedürfnisse Ihrer Schülerinnen und Schüler anpassen. Wie können Ihnen digitale Medien dabei helfen, dieses Ziel zu erreichen?“). Einige der Items beziehen sich auf spezifische Technologien (z. B. Tablets), während andere eher allgemeiner Natur sind (z. B. bezogen auf computerbasiertes Feedback). In einer Studie mit Studierenden wurden 14 Items identifiziert, die das latente Konstrukt von TPK mit einer zufriedenstellenden Modellpassung repräsentieren; allerdings konnte keine konvergente und diskriminante Validität festgestellt werden, wenn der Test mit Selbstberichtsmaßen in Beziehung gesetzt wurde: Die Ergebnisse im Wissenstest korrelierten positiv mit selbstberichtetem technologischem Wissen, aber nicht mit technologisch-pädagogischem oder pädagogischem Wissen. Der Test reagierte sensitiv auf pädagogische Interventionen, indem er signifikante Wissenszuwächse bei Lehramtsstudierenden aufzeigte, die eine Lehrveranstaltung zum Unterrichten mit digitalen Medien besucht hatten.

Die Vorhersagegüte der bislang entwickelten Tests hängt vermutlich damit zusammen, wie viel Unterrichtsbezug die Items aufweisen. Die Items der genannten Wissenstests sind eher abstrakt und losgelöst von der Unterrichtspraxis formuliert, um möglichst breit einsetzbar zu sein. Doch gerade Personen mit viel professioneller Erfahrung haben typischerweise Schwierigkeiten, das Wissen zu verbalisieren, das Grundlage ihrer Leistungsfähigkeit ist. Ihr Wissen ist in Handlungsskripte eingebunden, die ihr professionelles Verhalten leiten, die aber nicht mehr verbalisiert werden können (*encapsulated knowledge*, Boshuizen und Schmidt 1992; Pauli und Reusser 2003). Insbesondere für im Umgang mit digitalen Medien erfahrene Lehrpersonen könnten daher Testformate, die einen bewussten Zugang zum zugrunde liegenden Wissen erfordern, nicht genau das erfassen, was ihre Leistung vorhersagt. Vielmehr werden hier handlungsbezogenere und situierte Testformate erforderlich wie z. B. auf Vignetten basierende Items (z. B. Seidel und Stürmer 2014) oder rubrikenbasierte Bewertungen von Unterrichtsplänen (z. B. Harris et al. 2010).

Obwohl der Anspruch des TPACK-Modells gerade darin besteht, digitalisierungsbezogenes Wissen so zu beschreiben, dass es eine Vorhersage der Unterrichtsqualität ermöglicht, gibt es bislang nur wenige quantitative Studien zum Verhältnis der TPACK-Dimensionen und der Qualität von TET. Diese finden bislang keine Zusammenhänge, was allerdings durch die Güte der verwendeten Maße bedingt sein kann (z. B. Kopcha et al. 2014; Schmid et al. 2020).

Aufgrund des Fehlens effizienter Testinstrumente zur Erfassung des Wissens für das Unterrichten mit digitalen Medien basieren auch die bislang einzigen diesbezüglich verfügbaren umfassenden Datensätze aus TALIS (Teaching and Learning International Survey; OECD 2014) und ICILS (Information Computer Literacy International Study; Fraillon et al. 2019) lediglich auf Selbstberichten. Sie beschreiben daher eher Selbstwirksamkeitserwartungen von Lehrpersonen und nicht faktisches Wissen (Scherer et al. 2017). In TALIS (*N* = 50.800 Lehrpersonen; Deutschland nahm nicht teil) stimmten fast 70 % der Lehrpersonen zu, dass sie das Lernen ihrer Schülerinnen und Schülern durch den Einsatz von Technologie sehr oder ziemlich unterstützen können (Hämäläinen et al. 2021). Alter und Geschlecht waren mit diesen Selbstwirksamkeitsüberzeugungen assoziiert, wobei weibliche und ältere Lehrpersonen angaben, über geringere Fähigkeiten bzw. weniger Wissen zu verfügen. Drossel et al. (2019) diskutieren selbstberichtete digitale Kompetenzen von Lehrkräften auf Basis von Daten aus ICLIS 2018. Hier gaben 98,1 % der teilnehmenden Lehrkräfte in Deutschland an, nützliche Unterrichtsmaterialien im Internet finden zu können und 78,9 % sahen sich in der Lage, Unterricht unter Nutzung digitaler Technologien vorzubereiten. Diese Werte liegen über bzw. leicht unter dem internationalen Mittelwert aller an ICLIS 2018 teilnehmenden Länder. In Bezug auf spezifischere Technologienutzungen berichten die Lehrkräfte in Deutschland jedoch von Fähigkeiten, die weit unter dem internationalen und EU-Mittelwert liegen: Nur 49,3 % gaben an, Technologie zu diagnostischen Zwecken nutzen zu können (internationaler Mittelwert: 78,4 %) und 33,6 % sahen sich in der Lage, ein Lernmanagementsystem zu verwenden (internationaler Mittelwert: 58,8 %).

Während der Zusammengang zwischen digitalisierungsbezogenem Wissen und Unterrichtsqualität empirisch noch nicht etabliert wurde, gibt es erste Untersuchungen, nach denen motivationale Kompetenzaspekte einen Einfluss auf die Qualität des Unterrichts haben. Beispielsweise zeigten Backfisch et al. (2020) in einer quasiexperimentellen Expertisestudie, dass Lehrpersonen mit Unterrichtserfahrung (berufstätige Lehrkräfte sowie Referendare und Referendarinnen) qualitativ hochwertigere, mediengestützte Unterrichtsentwürfe im Bereich der Mathematik entwickelten als Lehramtsstudierende. Lehrpersonen mit Unterrichtserfahrung verfügten über ausgeprägteres pädagogisches Wissen und waren stärker von der Nützlichkeit digitaler Medien für den Unterricht überzeugt. Jedoch konnten nur diese höheren Nützlichkeitsüberzeugungen erklären, warum erfahrene Lehrpersonen bessere Unterrichtsentwürfe ablieferten. Die besondere Wichtigkeit von Nützlichkeitsüberzeugungen bestätigte sich in einer weiteren Studie mit berufstätigen Lehrpersonen, die aufgefordert wurden, Stunden, in denen sie Medien eingesetzt hatten, in einem Unterrichtstagebuch zu beschreiben (Backfisch et al. 2021). Diese Einträge wurden im Hinblick auf die instruktionale Qualität und den Innovationsgrad des Unterrichts ausgewertet. Beide Variablen wurden durch digitalisierungsbezogene Nützlichkeitsüberzeugungen, nicht aber durch entsprechende Selbstwirksamkeitserwartungen vorhersagt.

Im Vergleich zur Forschung zum Lernen mit digitalen Medien handelt es sich bei der Forschung zum Lehren mit digitalen Medien um ein vergleichsweises junges Feld, welches durch eine starke Lückenhaftigkeit und Fragmentierung gekennzeichnet ist. Auch wenn manche Bereiche wie z. B. das digitalisierungsbezogene Wissen von Lehrpersonen vor dem Hintergrund des TPACK-Modells (Mishra und Koehler 2006) durch eine Vielzahl von Publikationen gekennzeichnet sind, ist die damit verbundene Erkenntnis doch eher gering. Insbesondere ist immer noch offen, welche digitalisierungsbezogenen Unterrichtspraktiken zur einer höheren Unterrichtsqualität beitragen, die sich ihrerseits auf Lernprozesse und -ergebnisse der Schülerinnen und Schüler auswirkt und wie die Qualität medienbasierten Unterrichts durch die professionelle Kompetenz der Lehrpersonen mitbestimmt ist.

## Fazit und Ausblick

Salomon hat bereit 1990 gefordert, dass nicht ein einzelnes Forschungsperspektive die Beantwortung der Frage dominieren darf, wie und unter welchen Bedingungen digitale Medien Bildungsprozesse positiv beeinflussen können. Um hier wesentliche Erkenntnisfortschritte zu erzielen, ist es wichtig, sowohl die Flöte – einzelne Lernmedien – als auch das Orchester – mediengestützten Unterricht – zu untersuchen. „The music we enjoy is produced by symphonic orchestras, not just single flutes“ (Salomon 1990, S. 530). Bis zum heutigen Tag sind aber beide Forschungslinien weitestgehend unverbunden.

Das ist insofern überraschend, da sowohl die Forschung zum Lernen mit digitalen Medien (TEL) als auch die medienbezogene Unterrichtsforschung (TET) eine funktionale Betrachtung auf digitale Medien verfolgt, wonach ein Mehrwert dann resultiert, wenn Medien Lern- bzw. Lehrprozesse passgenau unterstützen. In beiden Forschungslinien wird davon ausgegangen, dass diese Funktionalität nicht an leicht zugänglichen Verhaltensmerkmalen von Lernenden (z. B. die Häufigkeit der Nutzung von Interaktionsmöglichkeiten in einer Animation) bzw. oberflächlichen Strukturmerkmalen des Unterrichts (Sichtstruktur; Kunter und Trautwein 2013) festgemacht werden kann. In der TEL-Forschung wird explizit zwischen behavioraler und kognitiver Aktivität unterschieden (Kennedy 2004; Mayer 2009) und darauf verwiesen, dass digitale Medien kognitive Aktivität (*minds-on*) und nicht notwendigerweise behaviorale Aktivität (*hands-on*) unterstützen sollten bzw. dass behaviorale Aktivität nicht notwendigerweise durch kognitive Aktivität begleitet wird. Das entspricht der Auffassung in der (medienbezogenen) Unterrichtsforschung, dass nur die Tiefenstruktur des Unterrichts Aufschluss über dessen Qualität erlaubt (Kunter und Trautwein 2013). Die Häufigkeit der Mediennutzung im Unterricht ist dabei lediglich ein Merkmal der Sichtstruktur.

Schließlich gibt es Gemeinsamkeiten hinsichtlich der Qualitätsmerkmale, anhand derer erfolgreiches Lernen bzw. Lehren mit digitalen Medien festgemacht werden kann. In der TEL-Forschung ist eine wesentliche Erkenntnis, dass digitale Medien so gestaltet sein müssen, dass verfügbare kognitive Ressourcen (z. B. Aufmerksamkeit, Gedächtniskapazität) auf lernbezogene Prozesse verwendet werden und nicht auf Prozesse, die für das Lernen irrelevant sind (z. B. die Suche nach lernzielbezogenen Informationen; *Cognitive Load Theory*, Sweller et al. 1998; *Cognitive Theory of Multimedia Learning*, Mayer 2009). Darüber hinaus benötigen Lernende zusätzliche instruktionale Unterstützung, um relevante Lernprozesse ausführen zu können (Belland et al. 2017). In kollaborativen computergestützten Lernumgebungen wird durch Scripting die Rollenverteilung der Lernenden und die Fokussierung auf die Aufgabenbearbeitung sichergestellt (Vogel et al. 2017). Gestaltungs- und Instruktionsmaßnahmen dienen dazu, dass Zeit und andere Ressourcen für das Lernen maximiert werden und erfüllen damit eine ähnliche Funktion wie eine effiziente Klassenführung im Unterricht (Kunter et al. 2007). Darüber hinaus erlauben digitale Medien durch die Vergabe individuellen Feedbacks eine konstruktive Unterstützung der Lernenden. Die größte Übereinstimmung von TEL-Forschung und Unterrichtsforschung ist bezogen auf Annahmen zur kognitiven Verarbeitung der Lernenden feststellbar. Beide Forschungslinien postulieren die Notwendigkeit, eine tiefergehende kognitive Verarbeitung des Lerninhalts anzuregen (Chi und Wylie 2014; Lipowksy et al. 2009). Kognitiv aktivierende Aufgaben sind aus Sicht der TEL-Forschung vor allem generative bzw. konstruktive Lernaufgaben, die Lernende dazu auffordern, über gegebene Information hinaus zu gehen (Elaboration) und ihr elaboriertes Wissen in externen Repräsentationen aufzubereiten (z. B. in Zeichnungen, als Selbsterklärungen oder Erklärungen für andere; vgl. Chi und Wylie 2014; Fiorella und Mayer 2016). Zusammenfassend können spezifische Affordanzen digitaler Medien auf Prozessmerkmale des Unterrichts abgebildet werden (siehe auch Stegmann 2020). So kann ein Unterricht mit adaptiven Technologien eine optimale kognitive Aktivierung der Lernenden ermöglichen, indem diese Lernaufgaben erhalten, die optimal an das aktuelle Fähigkeitsniveau angepasst sind. Außerdem können adaptive Lernangebote ein unterstützendes Klima fördern, indem sie individualisiertes Feedback und weitere Instruktionen zur Überwindung von Wissenslücken anbieten.

Bisherige Forschung nutzt diese Gemeinsamkeiten in den Grundannahmen von TEL und TET nicht aus. In der TEL-Forschung überwiegt die isolierte Betrachtung von Mediendesignmerkmalen ohne deren Untersuchung im Unterrichtsgeschehen, während in der TET-Forschung das Unterrichten mit digitalen Medien ohne Berücksichtigung medienspezifischer Affordanzen dominiert. Es erscheint hier aber vielversprechender, das Zusammenwirkungen von Mensch und Maschine im Sinne einer Aufgabenteilung bei der Gestaltung lernwirksamen Unterrichts zu betrachten. Danach sollten Lehrpersonen medienspezifische Affordanzen für das Lernen erkennen und beurteilen können sowie in ihrem Unterricht nutzen, während umgekehrt digitale Medien so gestaltet sein sollten, dass sie Lehrende in ihrem Unterrichtshandeln bestmöglich unterstützen.

Aus einer Zusammenführung von TEL und TET ergibt sich großes Potenzial für zukünftige Forschung an der Schnittstelle. Beispielsweise erlaubt sie es der Frage nachzugehen, inwieweit objektive Tests zur Erfassung digitalisierungsbezogenen Wissens aber auch diesbezügliche Selbsteinschätzungen valider werden, wenn sie auf – aus der TEL-Forschung bekannte – Affordanzen digitaler Medien bezogen sind. In dem am häufigsten genutzten TPACK-Instrument werden Lehrpersonen für die Erfassung von TPK z. B. gebeten einzuschätzen, ob sie in der Lage sind Technologien auszuwählen, die das Lernen von Schülerinnen und Schülern verbessern („I can choose technologies that enhance students’ learning for a lesson“, Schmid et al. 2009, S. 134). In ICILS 2018 (Fraillon et al. 2019) soll z. B. eingeschätzt werden, ob sich Lehrpersonen in der Lage sehen, mediengestützten Unterricht vorzubereiten. Diese vagen Itemformulierungen machen es notwendig, dass die Lehrperson zunächst für sich entscheiden muss, was lernwirksamer mediengestützter Unterricht eigentlich ist. Hier können faktisch wenig kompetente Lehrpersonen zu einer positiven Einschätzung gelangen, weil in ihrer Vorstellung guter mediengestützter Unterricht bereits in der Nutzung des Beamers zur Präsentation von Folien zum Ausdruck kommt. Umgekehrt wissen faktisch hoch kompetente Lehrpersonen vermutlich um den großen Handlungsspielraum, den digitale Medien bieten, und schätzen dementsprechend ihre eigenen Kompetenzen möglicherweise konservativer ein. Ebenso beziehen sich existierende Items oftmals entweder gar nicht auf konkrete Anwendungen und wenn dann nur auf generische Programme zur Textverarbeitung oder Informationssuche im Internet, nicht aber auf explizit für das Lernen spezifische Programme. Der Bezug auf in der TEL-Forschung untersuchte Medien und deren lernbezogene Affordanzen in Items zur Erfassung digitalisierungsbezogenen Wissens könnte daher die Messgüte dieser Items sowie deren Vorhersagekraft für die Qualität mediengestützten Unterrichts verbessern (Lachner et al. 2019).

Aus der TEL-Forschung sind zahlreiche Hinweise zur lernförderlichen Gestaltung digitaler Medien und teilweise auch konkrete Produkte hervorgegangen. Allerdings zeigen z. B. die Erfahrungen mit dem Webportal Golabz.eu, welches die weltweit größte Sammlung an Simulationen für Lernzwecke bereitstellt, dass Lehrpersonen insbesondere in Deutschland solche (forschungsbasiert entwickelten) Ressourcen nur relativ sporadisch nutzen (de Jong et al. 2021). Daraus ergibt sich Forschungsbedarf an der Schnittstelle von TEL und TET, wie Lehrpersonen – über die Bereitstellung von lernwirksam gestalteten Medien hinaus gehend – bei der Einbindung dieser Ressourcen in ihren Unterricht unterstützt werden können z. B. durch die forschungsbasierte Entwicklung von Unterrichtskonzepten für die Nutzung von Simulationen im Unterricht. De Jong et al. (2013) bezeichnen diese Identifikation von Fertigkeiten und Strategien zur Einbindung von Simulationen in den Unterricht als eine der großen Herausforderungen im Kontext forschenden Lernens, die auch Gegenstand entsprechender Fort- und Weiterbildungsprogramme sein muss.

Eine weitere von de Jong et al. genannte Herausforderung bezieht sich auf die Nutzung von Lernprozessdaten zur Gestaltung personalisierter Lernerfahrungen. Die TEL-Forschung verfügt über eine Vielzahl von prozessbezogenen Methoden und Befunden, die unter anderem für die Gestaltung adaptiver Lernumgebungen genutzt werden (Aleven et al. 2017). Allerdings sind solche Lernumgebungen nur eine Möglichkeit der adaptiven Gestaltung von Lernerfahrungen. Im Unterricht bietet sich darüber hinaus die Möglichkeit, Lernprozesse und -ergebnisse z. B. über so genannte Dashboards Lehrpersonen zugänglich zu machen, so dass die Daten für eine Umsetzung adaptiven Unterrichts genutzt werden können (e.g., Xhakay et al. 2017). Hier ergibt sich zukünftig großer Forschungsbedarf an der Schnittstelle zwischen TEL und TET z. B. dazu, welche Lernprozessdaten für Lehrpersonen informativ und nützlich sind, wie diese aufbereitet werden müssen oder wie Lern- und Unterrichtsprozesse so ineinandergreifen können, dass möglichst alle Schülerinnen und Schüler für sie optimale Lernmöglichkeiten erhalten. Auch hier stellt sich die aus TET-Sicht relevante Frage, welche Kompetenzen Lehrpersonen benötigen, um datenbasiert adaptiven Unterricht zu gestalten.
